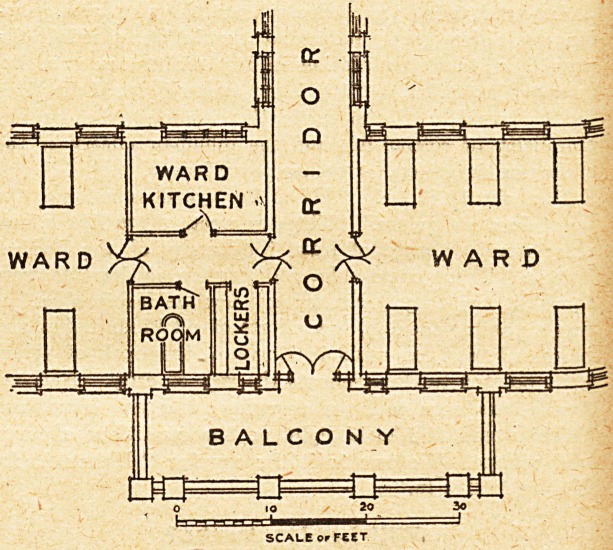# Articles on Hospital Construction

**Published:** 1919-03-01

**Authors:** 


### THE NEW ERA.

### III.—Articles on Hospital Construction.

Open-air treatment has happily taken such hold upon English Hospital authorities that proper provision for it can no longer be ignored by the designers of hospitals. It was no doubt the success of this method in tuberculous cases that led to the appreciation of the possibility of applying it to other than phthisical cases. There was prejudice to overcome both on the part of patients and on that of their attendants; but prejudice has been beaten and the provision of at least some accommodation which has a roof without four walls is universal throughout the kingdom. Sometimes it will be found that the open-air patients are permanently treated in their open-air shelter, in others the verandah or balcony or wall-less ward is only resorted to in the warmer hours of the day. This means that in some hospitals the open-air beds are counted as "beds," while in the others they are merely an occasional outside appearance of beds that are normally indoors.

We have remarked that the open-air ward has to be reckoned with and allowed for. Unfortunately, this feature is in many hospitals only accorded a sort of "poor relation" existence. It has been introduced half-heartedly as an unavoidable expedient, and treated like other poor relations as a nuisance both to himself and to his more established kinsmen. Any one with experience of hospitals will know what we mean.

A common plan has been to turn one of the windows of a ground-floor ward into a door, preferably on the south side (if there is a south side), and to build against the whole length of the ward a lean-to shed. A remnant of conscience on the part of the experimenters generally insists that a portion of the ward windows shall remain uncovered by the pent-roof, so that ventilation shall not be wholly extinguished. But even so, how unsatisfactory is the result. To be fair on the hospital architect it is only right to say that these shanties are often put up by the committee "behind his back." The result is lamentable. How can it be otherwise? The ward itself which needed its south windows has been deprived of half its light and ventilation, and is made not only less cheerful, but definitely less wholesome. For half the year in most parts of England the prevailing wind is south-west, and the current of air which crosses the ward consequently reaches the indoor patients (save for the bit of unrestricted window which may have been left at the top) across the beds of the outside patients, some of whom may have been given their outside berths for reasons which make this line of air-current specially unsatisfactory. We have seen a ward which, with its windows almost entirely blocked by such an intrusion, has become cheerless, sunless, and stuffy.

Again, it is sometimes taken for granted that any place is suitable for an open-air ward so long as the ward is deprived of the comforts of side shelter. This is a great mistake. To wedge in a sort of cart-shed in a place between high buildings, through which there passes a continual draught is not by any means a proper provision for patients. What is wanted is fresh air without draught and the maximum of sunshine without direct glare. These cannot be obtained without careful attention to aspect and situation.

In fact, the open-air ward wants designing just as much as any other part of the hospital's accommodation, and those architects who know what it is to be obliged to fit in such places as part of an already planned hospital are aware that the problem can be a very difficult one indeed.

It will sometimes be found that such wards can be satisfactorily erected on the flat roofs of other wards. It is not, of course, a good plan to make such accommodation open on all four sides, as there is in that case a liability for the wind to pass through too freely. A solid north wall, glazed sides which can be opened or closed at will, and an open south front from which driving rain can be excluded when necessary will provide the most satisfactory arrangement. If any part of this building has to abut on a ward care should be taken that its obstruction leaves the greater number of the ward windows perfectly free. Every ward, in an English hospital, generally has to put up with one unavoidable obstruction to light and air—the sanitary block. And when this has been placed in position where it will least hinder the access of sunshine and fresh breeze to the windows there is not, as a rule, much space left for the erection of the still larger obstruction offered by the open-air verandah. A plan which we publish herewith will show how the difficulty has been dealt with in a recent design.

☞ The previous articles appeared on January 25, p. 354, and February 8, p. 404.

## Figures and Tables

**Figure f1:**